# Interventions to Reduce Parental Substance Use, Domestic Violence and Mental Health Problems, and Their Impacts Upon Children’s Well-Being: A Systematic Review of Reviews and Evidence Mapping

**DOI:** 10.1177/15248380231153867

**Published:** 2023-02-15

**Authors:** Simon Barrett, Cassey Muir, Samantha Burns, Nicholas Adjei, Julia Forman, Simon Hackett, Raeena Hirve, Eileen Kaner, Rebecca Lynch, David Taylor-Robinson, Ingrid Wolfe, Ruth McGovern

**Affiliations:** 1 Newcastle University, UK; 2 Durham University, UK; 3 University of Liverpool, UK; 4 King’s College London, UK; 5 University of Exeter, UK

**Keywords:** alcohol and drugs, family issues and mediators, child abuse, cultural contexts, intervention/treatment, domestic violence, mental health and violence

## Abstract

**Introduction:**

Children exposed to parental intimate partner violence and abuse, mental illness, and substance use experience a range of problems which may persist into adulthood. These risks often co-occur and interact with structural factors such as poverty. Despite increasing evidence, it remains unclear how best to improve outcomes for children and families experiencing these adversities and address the complex issues they face.

**Aims and Methods:**

Systematic review of systematic reviews. We searched international literature databases for systematic reviews, from inception to 2021, to provide an evidence overview of the range and effectiveness of interventions to support children and families where these parental risk factors had been identified.

**Results:**

Sixty-two systematic reviews were included. The majority (*n* = 59) focused on interventions designed to address single risk factors. Reviews mostly focused on parental mental health (*n* = 38) and included psychological interventions or parenting-training for mothers. Only two reviews assessed interventions to address all three risk factors in combination and assessed structural interventions. Evidence indicates that families affected by parental mental health problems may be best served by integrated interventions combining therapeutic interventions for parents with parent skills training. Upstream interventions such as income supplementation and welfare reform were demonstrated to reduce the impacts of family adversity.

**Conclusion:**

Most intervention approaches focus on mitigating individual psychological harms and seek to address risk factors in isolation, which presents potentially significant gaps in intervention evidence. These interventions may not address the cumulative impacts of co-occurring risks, or social factors that may compound adversities.

## Introduction

It is estimated that between 2.1 and 5.4 million children in England live in homes with at least one parental risk factor of intimate partner violence and abuse (IPVA), mental illness, or substance use (Children’s Commissioner, 2018). In addition to well-documented harms to the parent due to these risk factors (Rehm & Shield, 2019; World Health Organization, 2013), children exposed to these risk factors experience a greater range of problems which may emerge in early years and persist into adulthood (Adjei et al., 2021). Such children are more likely to suffer accidental injury (Yang et al., 2020), ill-health, and encounter barriers to access appropriate care for their health needs (Artz et al., 2014), and have lower educational performance (Cleaver et al., 2011), resulting in poor life outcomes (Artz et al., 2014). Children exposed to family adversities are more likely to experience mental health problems themselves (Grip et al., 2012), while children who live in households where IPVA occurs additionally experience trauma symptoms (Evans et al., 2008; Grip et al., 2012). Children exposed to each of these adversities are more likely to engage in health compromising behaviors such as substance use and engage in anti-social behavior (R. McGovern et al., 2018; Whitaker et al., 2006). These children may experience multiple disadvantages in adulthood, including poor employment opportunities, lower incomes, poor physical and mental health, problematic substance use, and offending behavior (Goodman et al., 2011). Additionally, children who are exposed to IPVA are more likely to later be victim to, or perpetrate, interpersonal violence (Murrell et al., 2007). These risks co-occur with cumulative impact (Whitaker et al., 2006) and are themselves driven and exacerbated by structural risk factors such as poverty (Adjei et al., 2021).

## Mechanisms of Impact

Direct exposure to IPVA (Haselschwerdt et al., 2019), parent’s alcohol and/or drug use and/or to other substance users (Advisory Council on the Misuse of Drugs, 2003), or parent’s mental health symptoms (Manning & Gregoire, 2006) have all been linked to harmful impacts on child health and well-being. IPVA has been found to negatively affect the structures and functions of the family and relationships between adult and child victims (Bancroft et al., 2011). While some studies suggest mothers who experience IPVA may demonstrate increased warmth and responsiveness toward their children (Austin et al., 2019), others indicate they may be less able to respond to the emotional needs of their children (Levendosky & Graham-Bermann, 2001). This may be driven by fear they experience within the home, as well as negative social, emotional, and physical health consequences of victimization (Lapierre, 2008). Within families affected by parental substance use, punitive parenting practices, and a reduction in parenting capacity brought about by the intoxicating effect substances and/or withdrawal are reported (Kandel, 1990; Miller et al., 1999), alongside a potential lack of parental emotional availability and warmth (Suchman et al., 2007).

A recent study indicated that over 40% of children in the UK Millennium Cohort experienced continuous exposure to either poor parental mental health and/or poverty and these common exposures were associated with large negative impacts on child physical, mental, cognitive, and behavioral outcomes (Adjei et al., 2021). Parents’ psychological problems may lead to negative parenting behaviors, lack of attention to children’s needs, or increased dysfunction within the home influencing attachment and impacting early child development (Wilson & Durbin, 2010). Further, harm may be direct, with children living in households where one or more of these parental risk factors are present being more likely to experience child maltreatment (Dube et al., 2001); or indirect where children worry about parents’ welfare, or through insecurity brought about by separation from parents during periods of hospitalization (Manning & Gregoire, 2006), or incarceration (Travis et al., 2014). The stigma surrounding each of these risk factors, as well as the lack of availability of support services for affected families, may also contribute to the difficulties and complexities faced by caregivers when caring for their children (W. McGovern et al., 2022; Muir et al., 2022).

## Clustering of Adversity and Syndemic Risks

Importantly, these childhood adversities are known to co-occur or cluster (Lacey et al., 2022; Lanier et al., 2018), and there is increasing evidence that poverty is a strong reinforcing factor in the clustering and accumulation of adversity (Bywaters et al., 2022; Lacey et al., 2022; Walsh et al., 2019). Syndemics is a relatively recent concept which provides a framework for understanding health conditions that arise in populations. Syndemic approaches recognize that health conditions can be formed, and/or exacerbated, by the social, economic, environmental, and political milieu in which the population is immersed, and describes the presence of two or more conditions that adversely interact with each other, negatively affecting the mutual course of the trajectory of each. Syndemic interactions enhance vulnerability and are made more harmful by experienced inequities (The Lancet, 2017). For example, parental mental health problems have been demonstrated to interact syndemically with structural risk factors such as poverty across childhood developmental stages, with large negative impacts on health outcomes and behavior in later life (Adjei et al., 2021).

## How Interventions May Work to Reduce the Impact

Interventions to reduce the impact of parental risk factors such as IPVA, mental illness, or substance use may target individuals, dyads, or families, or they may attempt to affect changes at the population or system level. Interventions may take the form of primary prevention focused on improving family support and reducing exposure to childhood adversities and their determinants, or secondary prevention which seeks to mitigate the impacts of these determinants. Interventions may directly target parents and aim to reduce the risk factor(s), or alternatively parents may be supported to develop their parenting skills to moderate impacts upon children, either through promoting better attachment and reflective functioning, or through broader skill development. Interventions may work with parents and children jointly, or children may be the focus of interventions designed to help them cope with adversities. Such approaches may seek to build resilience, address trauma, or provide social support. It is important to note that while parents may individually or together experience poor mental health and/or substance use, IPVA often involves one parent harming another. This creates a complex situation for children who may themselves need protection, but may also wish to protect the victim parent and preserve their own relationship with the perpetrator.

## Study Objectives

To date, there have been multiple systematic reviews of the effectiveness of interventions to reduce parental risk factors (R. McGovern, Newham, et al., 2021; Moreland & McRae-Clark, 2018; Niccols et al., 2012; Siegenthaler et al., 2012), improve parenting practices in the context of risk factors (Austin et al., 2019; Barlow et al., 2003), or intervene with affected children to reduce adversity (Calhoun et al., 2015; Havinga et al., 2021). However, no review has engaged with the syndemic nature of these problems, to understand what is known about how best to respond to the complex and interconnected issues experienced by vulnerable families. Much is already known about pharmacological approaches for single risk factors (see Cipriani et al., 2018) and therefore our focus is upon psychosocial strategies which intervene with individual or combined risk factors. It is unclear if or what combination of individual, population, or system-level interventions are likely to be effective at reducing the impact of parental risk factors identified within families, or which of these interventions may offer the greatest opportunity to improve outcomes for children and other family members in the presence of these risk factors. This systematic review of reviews therefore examines what is currently known about non-pharmacological interventions which aim to reduce levels of IPVA, parental mental illness, and parental substance use, either individually or in combination, and the effectiveness of interventions to support children exposed to these risk factors. We also examine experiences of these interventions. We have used an evidence overview approach to map this complex field of interventions to address overlapping risks which are targeted at differently affected family members, and to identify key gaps which need to be filled.

## Methods

The review protocol was registered with the International Prospective Register of Systematic Reviews in January 2021 (PROSPERO; Registration Number: CRD42021233785).

International literature was searched from inception to April 2021 using the following electronic databases: MEDLINE (OVID), PsycINFO (OVID), Applied Social Science Index and Abstract (ProQuest), International Bibliography of Social Science (ProQuest), ProQuest Social Science Journals, ProQuest Sociology, Social Service Abstracts (ProQuest), Sociological Abstracts (ProQuest), and EBSCO. A search strategy using key terms, thesaurus headings, Boolean, and proximity operators was adapted and implemented for each database. No language or geographical restrictions were applied.

### Review Inclusion Criteria

Two researchers independently screened all titles and abstracts using specified inclusion and exclusion criteria, retrieving full papers for all potentially eligible studies and evaluating full text. Discrepancies at each stage were resolved by discussion or by consulting a third researcher if consensus could not be reached. We included systematic reviews of primary studies, which we defined as reviews which described explicit and reproducible methods to systematically search and synthesize data. These reviews incorporated: outcome evaluations, randomized controlled trials, controlled trials and randomized trials, quasi-experimental designs, and qualitative studies. Reviews were included if they provided non-pharmacological intervention to families, or parents and/or children (aged 0–18 years or up to 25 years for care leavers) where parental risk factors of IPVA, mental illness, and/or substance use have been identified.

Data extraction was completed using a bespoke, piloted data extraction form, where key characteristics of each of the reviews were recorded, including details of the risk factor of focus, study type, the level and target populations of interventions, and the main findings of each review. The results of our review are narratively synthesized, and reported in response to each of our review questions: what is currently known about interventions which aim to reduce levels of IPVA, parental mental illness, and parental substance use identified in families; what is the evidence for the effectiveness of interventions which support children exposed to these risk factors; and how are these interventions experienced by participants?

This approach allowed us to consider the effectiveness and experiences of a broad range of interventions, delivered at differing levels to a variety of participants. Due to the fact that there may be some overlap of individual trials within the included reviews, we focus on the overall meta-analytical results and summative outcomes to provide an evidence overview, rather than reporting on individual trial conclusions. We report overall effect sizes wherever available, using the standardized mean difference (SMD); SMDs of 0.2, 0.5, and 0.8 are considered small, medium, and large, respectively (Andrade, 2020). Due to potential double-counting, the heterogeneous nature of the literature, and outcomes measured and tools used across these studies, meta-analysis was not possible.

### Quality Appraisal

The methodological quality of each study included was assessed according to the criteria outlined in the Joanna Brigg’s Institute Critical Appraisal Checklist for Systematic Reviews and Research Syntheses tool (Aromataris et al., 2015). This tool addresses 11 domains, with an option of yes, no, or unclear/not applicable. The results of this appraisal informed the synthesis and the interpretation of results and can be found in the Supplemental Material. Due to the heterogeneous nature of the reviews included, it is not appropriate to form an overall score or summative assessment of study quality. However, all included reviews reported their search strategy, their inclusion criteria, and their methods and sources. The vast majority (85%) included a clearly stated aim. Only 67% made recommendations for policy or practice, and 56% were found to have adequately assessed potential publication bias.

## Results

### Description of Reviews

**Figure 1.**
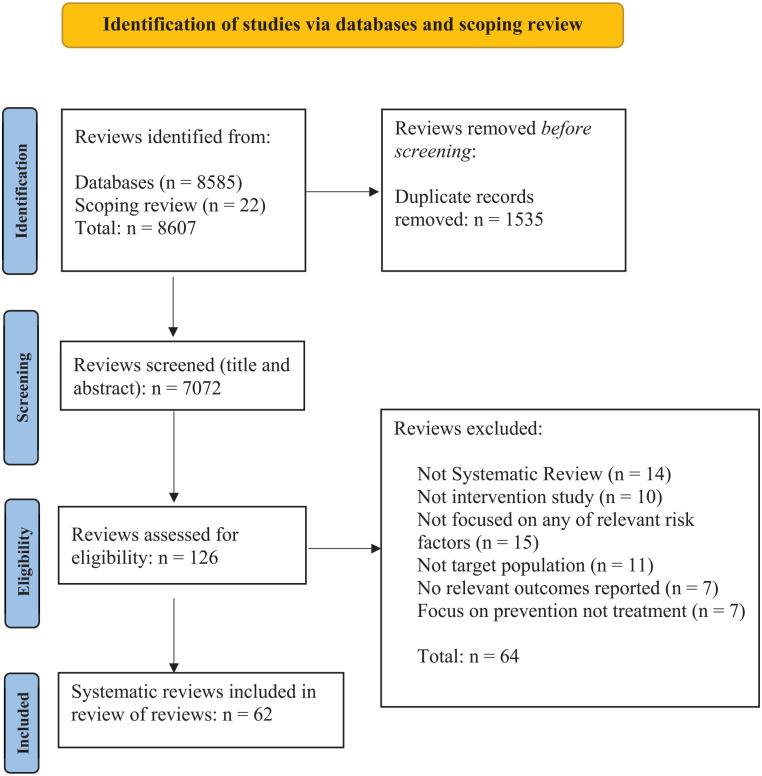
PRISMA flow chart.

Sixty-two published systematic reviews met our inclusion criteria. Figure 1 shows a PRISMA flow diagram for the identification and inclusion of studies.

Fifty-nine reviews (95%) reported on interventions addressing a singular parental risk factor, with the majority examining interventions for parental mental health problems (*n* = 38; 61%). Parental substance use was examined in 14 reviews (23%), and IPVA in 7 reviews (11%). One review (2%) included two parental risk factors in combination (parental substance use and IPVA). A further two reviews (3%) included all three parental risk factors of IPVA, mental health problems, and substance use (Courtin et al., 2019; Marie-Mitchell & Kostolansky, 2019). Table 1 provides a descriptive summary of the included reviews, alongside critical findings, and intervention effect sizes where reported (SMD and 95% confidence intervals (CIs)).

**Table 1.** Summary of Critical Findings, with Effect Sizes Using Standardized Mean Differences (SMD) and 95% Confidence Intervals (CIs) Where Reported.

Author (Year) (No. Studies in Review)	Risk Factor(s) Targeted	Intervention Recipient	No. of Participants in Review	Intervention Type	Review Outcomes and Critical Findings (Effect Sizes [SMD] and 95% CIs, Where Reported)
Effectiveness of interventions at reducing parental mental health problems					
Daley et al. (2009) (5)	Mental health	Mother	221	Psychoeducational	Some reductions in postnatal depression: −0.81, [−1.53, −0.10].
Hanach et al. (2021) (10)	Mental health	Mother	2,366	Cognitive behavioral therapy (CBT)	Effective at reducing postnatal depression: −1.81, [−2.68, −0.93].
Leger and Letourneau (2015) (6)	Mental health	Mother	720	Psychoeducational	Mixed findings on reductions in postpartum depression.
Morrell (2006) (31)	Mental health	Mother	8,173	Psychoeducational	Limited evidence on effectiveness for preventing or reducing postnatal depression.
Leis et al. (2009) (6)	Mental health	Mother	1,151	CBT	Reductions in postnatal depression.
Goldstein et al. (2020)* (14)	Mental health	Father	—	Parenting skills/training	Mixed effectiveness for reductions in paternal perinatal depression.
Nillni et al. (2018) (73)	Mental health	Mother	—	CBT and interpersonal therapy (IPT)	Reductions in postnatal depression, anxiety, and trauma.
Song et al. (2015) (13)	Mental health	Mother	—	Psychoeducational	Effective at reducing postpartum stress: −1.66, [−2.74, −0.57].
Lapp et al. (2010) (7)	Mental health	Mother	—	CBT	Limited evidence on effectiveness for reducing posttraumatic stress disorder after childbirth.
Rominov et al. (2016)* (11)	Mental health	Father	1,730	Parenting skills/training	Mixed effectiveness for reductions in paternal perinatal depression.
Shi and MacBeth (2017) (17)	Mental health	Mother	603	Psychoeducational	Reductions in perinatal anxiety, mixed findings for perinatal depression.
MacBeth et al. (2015) (9)	Mental health	Mother-child dyad	95 treat/55 comparison	Parenting skills/training	Effective at reducing maternal mental health problems: −0.20, [−0.34, −0.06].
Kim and Lee (2019) (10)	Mental health	Mother	798	Psychoeducational	Effective at reducing mental health problems for low-income mothers with young children: −0.28, [−0.45, −0.11].
Reuveni et al. (2020) (6)	Mental health	Mother	—	IPT	Reductions in parental mental health problems.
Nair et al. (2018) (10)	Mental health	Mother	1,138	CBT	Some effectiveness at reducing maternal depression. Effect sizes ranged from −0.69 to −4.03.
Alexander (2018) (7)	Mental health	Parental dyad	775	Psychoeducational	Reductions in parental depression.
Barlow et al. (2012) (–)	Mental health	Parental dyad	4,937	Parenting skills/training	Effective at reducing parental depression: −0.17, [−0.28, −0.07]; and anxiety: −0.22, [−0.43, −0.01].
Huang et al. (2020) (13)	Mental health	Mother–child dyad	1,141 dyads	Parenting skills/training	Effective at reducing maternal depression: −0.25, [−0.40, −0.09] at short term follow-up but not long term.
Olhaberry et al. (2013) (27)	Mental health	Mother–child dyad	3,949+	CBT	Reductions in maternal depression.
Stiawa et al. (2014) (8)	Mental health	Family	—	Home visitation and financial support	Reductions in parental mental health problems.
Courtin et al. (2019) (28)	Mental health, substance use, IPVA	Family	540–15,376 households; 1,420–61,662 children	Socioeconomic interventions	Effect sizes were modest for household mental illness
Niccols et al. (2010)*** (5)	Substance use	Mothers	451	Integrated	Integrated interventions for maternal substance use reduction show improvements in maternal mental health outcomes: 0.23, [0.15, 0.31].
Stevenson et al. (2010) (6)	Mental health	Mother	672	CBT	Limited evidence on effectiveness for reducing postnatal depression.
Barlow et al. (2021) (6)	Mental health	Parent–child dyad	521	Parenting skills/training	Not effective for reducing parental depression.
Leijten et al. (2019) (10)	Mental health	Mother–child dyad	1,280 children	Parenting skills/training	Not effective at reducing maternal depression.
Leonard et al. (2021)** (13)	Mental health	Mother	5,540	Home visitation	Not effective at reducing maternal depression.
Effectiveness of interventions at reducing child mental health or behavioral problems, or improving child well-being					
Havinga et al. (2021) (8)	Mental health	Children	1,325	CBT	Effective at reducing children’s mental health problems: −0.19, [−0.36, −0.02].
Loechner et al. (2018) (7)	Mental health	Children	2,029	CBT	Effective at reducing children’s depression: −0.20, [−0.34, −0.06].
Basogul and Buldukoglu (2015) (3)	Mental health	Family	249 families	Parenting skills/training and psychoeducational	Reductions in children’s mental health problems.
Thanhäuser et al. (2017) (50)	Mental health	Parents and children	1,445 mother-infant dyads, 3,020 children	CBT and IPT	Effective at reducing children’s mental health problems: global psychopathology (*ES* = 0.13), internalizing symptoms (*ES* = 0.17).
MacBeth et al. (2015) (9)	Mental health	Mother–child dyad	95 treat/55 comparison	Parenting skills/training	Effective at reducing child behavioral problems: −0.40, [−0.77, −0.02].
Lindstrom Johnson et al. (2018) (21)	IPVA	Parent–child	1,361 children	Parenting skills/training	Effective at reducing children’s internalizing: 0.59, [0.43, 0.74], and externalizing: 0.48, [0.34, 0.62] problems.
Siegenthaler et al. (2012) (13)	Mental health	Parents and children	2,894	CBT	Effective at reducing children’s internalizing: −0.22, [−0.37, −0.08] and externalizing: −0.16, [−0.36, 0.04] problems.
Leijten et al. (2019) (10)	Mental health	Mother–child dyad	1,280 children	Parenting skills/training	Reductions in child conduct problems.
Marie-Mitchell and Kostolansky (2019) (20)	Mental health, substance use, IPVA	Family	—	Multiple family interventions	Reductions in child behavioral and mental health problems and improvements in parent−child relationships.
Calhoun et al. (2015) (4)	Substance use	Families	380	Integrated	Improvements in well-being of children. Effect sizes ranged from 0.20 to 0.50.
Peisch et al. (2018) (17)	Substance use	Parent–child dyad	483	Parenting skills/training	Mixed evidence on effectiveness for parent–child interaction and children’s behavioral/developmental outcomes.
Fraser et al. (2006) (26)	Mental health	Children	—	Psychoeducational	Limited evidence of effectiveness for improvements to children’s well-being.
Rizo et al. (2011) (31)	IPVA	Families	4,174	Parenting skills/training	Some improvements in children’s well-being.
Austin et al. (2019) (19)	IPVA	Mother–child dyad	1,551	Parenting skills/training	Uncertain on effectiveness for improving well-being among children
Effectiveness of interventions at reducing IPVA					
Ryan and Romam (2019) (6)	IPVA	Family	—	Parenting skills/training	Reduction in incidents of IPVA (as well as improvements in mother and child well-being).
Bilukha et al. (2005) (–)	IPVA	Family	—	Home visitation	Uncertain on effectiveness in reducing IPVA.
R. McGovern, Smart, et al., (2021) (58)	Substance use	Family	5,955	Parenting skills/training	Behavioral interventions were found to be effective in reducing intimate partner violence, as well as enhancing family functioning.
Giusto and Puffer (2018) (9)	Substance use, IPVA	Fathers	6,104	Psychoeducational	Reductions in IPVA as well as some improvements in substance use.
Courtin et al. (2019) (28)	Mental health, Substance use, IPVA	Family	540–15,376 households; 1,420–61,662 children	Socioeconomic interventions	Effect sizes were moderate to high for exposure to domestic violence.
Effectiveness of interventions at reducing parental substance use					
R. McGovern, Newham, et al., (2021) (22)	Substance use	Parental dyad	2,274	Integrated	Effective at reducing alcohol use: −0.56, [−0.96, −0.16] and drug use: −0.39, [−0.75, −0.03].
Milligan (2010)*** (21)	Substance use	Mother	—	Integrated	Effective at reducing maternal alcohol use: 0.40, [−0.31, 0.48] and drug use: 0.65, [0.57, 0.74].
Moreland and McRae-Clark (2018) (15)	Substance use	Mother	1,046	Integrated	Reductions in maternal substance use.
Giusto and Puffer (2018) (9)	Substance use, IPVA	Fathers	6,104	Psychoeducational	Some improvements in substance use, as well as reductions in IPVA.
Courtin et al. (2019) (28)	Mental health, substance use, IPVA	Family	540–15,376 households; 1,420–61,662 children	Socioeconomic interventions	Effect sizes were strong for substance abuse.
Effectiveness of interventions at improving parenting skills/family functioning					
Lindstrom Johnson et al. (2018) (21)	IPVA	Parent–child dyad	1,361 children	Parenting skills/training	Effective at improving parenting skills: 0.72, [0.43, 1.00].
Austin et al. (2019) (19)	IPVA	Mother–child dyad	1,551	Parenting skills/training	Uncertain on effectiveness for improving parenting skills amongst mothers exposed to IPVA.
Niccols et al. (2012)*** (4)	Substance use	Mothers	590	Integrated	Improvements in parenting skills, effect sizes ranged from −0.02 to 0.94.
Basogul and Buldukoglu (2015) (3)	Mental health	Family	249 families	Parenting skills/training and psychoeducational	Improvements in parenting skills.
Jidong et al. (2021) (13)	Mental health	Mother	—	CBT and parenting skills/training	Improvements in parenting skills.
Rizo et al. (2011) (31)	IPVA	Family	4,174	Parenting skills/training	Improvements in parenting skills.
Tsivos et al. (2015) (19)	Mental health	Mother	752	Parenting skills/training	Improvements in parenting skills.
Krahn et al. (2018) (4)	Substance use	Mothers	674 parents, 311 children	Parenting skills/training	Improvements in parenting skills.
Bowie (2004) (10)	Substance use	Mother–child dyad	961	Parenting skills/training	Mixed effectiveness at improving parenting skills.
Letourneau et al. (2017) (36)	Mental health	Mother–child dyad/mother	185 parent–child, 4,800+ women	Parenting skills/training	Mixed findings on effectiveness at improving parenting skills and child development.
Barlow et al. (2016) (8)	Mental health	Parent–child dyad	846	Parenting skills/training	Limited evidence of effectiveness (multiple effect sizes reported) for parent–child relationships.
Huang et al. (2020) (13)	Mental health	Mother–child dyad	1,141 dyads	Parenting skills/training	Limited evidence of effect on parent–child relationships.
Rayce et al. (2020) (7)	Mental health	Mother	758	Parenting skills/training	Not effective at improving parent–child relationships.
R. McGovern, Smart, et al., (2021) (58)	Substance use	Family	5,955	Parenting skills/training	Behavioral interventions were found to be effective in enhancing family functioning, as well as reducing intimate partner violence.
Calhoun et al. (2015) (4)	Substance use	Families	380	Integrated	Improvements in family functioning. Effect sizes ranged from 0.20 to 0.50.
Murphy et al. (2017) (11)	Substance use	Families	3,405 treat/3,304 control	Integrated	Positively associated with the likelihood of family reunification.
Barlow et al. (2021) (6)	Mental health	Parent–child dyad	521	Parenting skills/training	Moderate improvement in parental reflective functioning: −0.46, [−0.97, 0.04].
Courtin et al. (2019) (28)	Mental health, substance use, IPVA	Family	540–15,376 households; 1,420–61,662 children	Socioeconomic interventions	Effect sizes were modest for adverse parenting

*Note.* IPVA = intimate partner violence and abuse.

* Goldstein et al. (2020) and Rominov et al. (2016) are linked reviews.

** Leonard et al. (2018) and Leonard et al. (2021) are linked reviews.

*** Milligan (2010), Niccols et al. (2010), and Niccols et al. (2012) are linked reviews.

Most reviews (63%) reported on interventions delivered to mothers only (*n* = 29 mental health problems; *n* = 9 substance use; and *n* = 0 IPVA) or included parents regardless of gender (*n* = 10 mental health problems; *n* = 8 substance use; and *n* = 6 IPVA), although these reviews typically reported a majority maternal sample. A minority of reviews (6%) focused upon identified paternal risk factors (*n* = 2 mental health problems; *n* = 1 substance use; and *n* = 3 IPVA).

Around half of reviews reported on interventions targeted toward an individual (*n* = 33), with a parent being the main recipient of interventions, (*n* = 21 mental health problems; *n* = 7 substance use; and *n* = 1 IPVA), with only four reviews examining interventions targeting children (*n* = 3 mental health problems; *n* = 1 IPVA). A further 29 reviews focused on dyads or whole families as recipients of interventions (*n* = 15 mental health; *n* = 9 substance use; and *n* = 7 IPVA). The majority of interventions were from high income, anglophone countries: USA (325 interventions across 38 reviews); Australia (86 interventions across 15 reviews); UK (79 interventions across 25 reviews); and Canada (31 interventions across 18 reviews). Reported sample sizes ranged from 9 individuals to 3,371,454 households. Table 2 provides a gap map of the interventions showing the number of reviews by parent (mother/father/parent) or child across the different parental risk factors.

**Table 2.** Evidence and Gap Map of Interventions Showing **Total** Number of Reviews and by Parent (M = mother, F = father, P = parent) or Child (C) Outcomes Across Different Parental Risk Factors: Parental Mental Health Problems (MH), Parental Substance Use (SU), and Intimate Partner Violence and Abuse (IPVA).

	Parental Risk Factors						
	MH	IPVA	SU	MH/SU	MH/IPVA	SU/IPVA	All
Interventions							
Psychological and supportive interventions delivered to the individual parent to reduce the parental risk factor(s)							
CBT/IPT	**9** (9M)	—	—	—	—	—	—
Psycho-educational	**6** (5M/1P)	—	—	—	—	**1** (1F)	—
Psycho-social	—	—	**1** (1P)	—	—	—	—
Parenting skills and training	**8** (4M/2F/2P)	**1** (1P)	—	—	—	—	—
Integrated	—	—	**4** (2M/2P)	—	—	—	—
Home visits	**2** (1M/1P)	**1** (1P)	—	—	—	—	—
Parenting interventions to improve parenting capacity/relationships							
Parenting skills and training	**10** (7M/3P)	**5** (2M/3P)	**4** (2M/2P)	—	—	—	—
Integrated	—	—	**4** (2M/2P)	—	—	—	—
Psychological and supportive interventions for children affected by parental risk factors							
CBT/IPT	**4** (4C)	—	—	—	—	—	—
Psycho-educational	**2** (2C)	—	—	—	—	—	—
Parenting skills and training	—	**1** (1C)	—	—	—	—	—
Family-based	—	—	—	—	—	—	**1** (1C)
System	—	—	—	—	—	—	**1** (1C)
Experiences of interventions							
Reduce parental risk	**2** (2M)	—	**1** (1M)	—	—	—	—
Improve parenting	—	—	**1** (1P)	—	—	—	—
Support children	—	**1** (1C)	—	—	—	—	—

*Note.* Totals do not add up to 62 as some reviews have outcome data across more than one intervention or family member. CBT = cognitive behavioral therapy; IPT = interpersonal therapy.

### Psychological and Supportive Interventions Delivered to the Individual Parent

#### Parental Mental Health

Nineteen reviews reported on psychological or supportive interventions; almost all of which examined interventions for mothers during the postnatal period or with parents of infants/toddlers. Reviews of psychological therapies reported reductions in postnatal depression (PND) from cognitive behavioral therapy (CBT) (Hanach et al., 2021; Leis et al., 2009; Nair et al., 2018; Nillni et al., 2018) and interpersonal therapy (IPT) (Nillni et al., 2018; Reuveni et al., 2020), with SMDs for the effects ranging between −0.69 and −4.03 (see Table 1 for more detail). Additionally, reviews reported evidence of effect upon anxiety and trauma in the postnatal period from CBT (Nillni et al., 2018) and from IPT (Nillni et al., 2018; Reuveni et al., 2020). These reviews included interventions delivered in person (Leis et al., 2009; Nillni et al., 2018) and as telemedicine (Hanach et al., 2021; Nair et al., 2018; Nillni et al., 2018), with both approaches showing significant positive effects, which were sustained at follow-up. There were however concerns regarding the certainty of evidence of the effectiveness of CBT for parental mental illness within the postnatal period, largely due to lack of rigor in evaluation methods (Lapp et al., 2010; Nillni et al., 2018; Stevenson et al., 2010). Further, one review (Nillni et al., 2018) examined CBT and IPT with a focus on low-income and/or minority women with mixed results. One review examined psychological interventions, including CBT and IPT, for maternal depression specifically among women of African and Caribbean origin living in high-income countries (Jidong et al., 2021). The review reported interventions designed to enhance parenting confidence and self-care were effective, and the authors suggested that such interventions should also be culturally adapted and rigorously tested.

Most reviews providing psychological or supportive interventions to parents who experience mental health problems examined intervention effects upon the mental health of the parent. The only review to examine family and/or child outcomes (Olhaberry et al., 2013) concluded that cognitive behavioral models appeared to provide suitable and effective alternatives for reducing maternal depressive symptoms but not necessarily for improving the mother–infant bond.

Nine reviews examined psychoeducational interventions including symptom management, peer support, mindfulness, and exercise-based interventions. All of these reviews examined interventions that were entirely or mostly delivered within the postnatal period, with most reporting that these interventions positively impacted upon parental mental illness, with mixed effect sizes (see Table 1) (Alexander, 2018; Daley et al., 2009; Kim & Lee, 2019; Leger & Letourneau, 2015; Morrell, 2006; Shi & MacBeth, 2017; Song et al., 2015).

A meta-analysis undertaken by Leonard et al. (2021) examined the effectiveness of home visiting for maternal mental illness across eight randomized controlled trials. The findings indicated that home visiting was not effective in reducing either maternal depression or maternal stress. Stiawa et al. (2014) also reported on home visitation support and financial support interventions for families affected by parental mental health. Their review concluded that overall, such interventions were effective in significantly reducing symptoms of depression and anxiety and strengthening social skills, but only with temporary effectiveness. However, not all of the included interventions incorporated a financial support element, and those which did were based in the United States, and consisted of short-term, time-limited financial support or financial aid for medical care.

#### Intimate Partner violence and abuse

Two reviews examined interventions to reduce IPVA. Giusto and Puffer (2018) reported that psychoeducational interventions delivered to fathers, specifically those involving approaches which challenged gender norms, were effective at reducing IPVA. Common components of these interventions were structured discussion, goal-directed feedback, and psychoeducation targeting specific aims. Rizo et al. (2011) examined interventions that sought to address children’s needs through direct or indirect services. Only one intervention was offered to fathers who were perpetrators of IPVA and included goals such as ending IPVA and increasing fathers’ understanding of the impact of IPVA on children. Findings showed post-intervention reductions in hostility; denigration and rejection of child(ren); and angry arousal to child and family situations. The authors concluded that more research is needed with fathers, and perpetrators of IPVA.

#### Parental Substance Use

Two reviews examined psychosocial interventions for the substance using parent. A meta-analysis of eight randomized controlled trials found that interventions which only target the substance use of the parent were not effective at reducing the frequency of use (R. McGovern, Newham, et al., 2021). A further review examined psychoeducational interventions to address father’s alcohol use and reported six of the nine studies documented modest improvements in the level of drinking (Giusto & Puffer, 2018).

#### Parenting Interventions

##### Parental Mental Health

Ten reviews (reported in 10 published manuscripts) examined the effectiveness of parenting interventions to reduce parental mental health problems (Barlow et al., 2003, 2012, 2021; Goldstein et al. 2020; Huang et al., 2020; Jidong et al., 2021; Leijten et al., 2019; MacBeth et al., 2015; Rominov et al., 2016; Tsivos et al., 2015). Interventions largely included behavioral and cognitive behavioral components, as well as some that were classified as multi-modal, and were predominantly provided to mothers of young children and children with neurological difficulties (such as autism and attention deficit and hyperactivity disorder). Whilst these reviews mostly reported positive intervention effect in the short term, these effects were either not reported or not sustained at longer-term follow-up. Barlow et al. (2012) also suggested that despite insufficient evidence to clearly demonstrate an impact on paternal psychosocial functioning, the limited evidence available did suggest that parenting programs had potential to do so, and as such the authors called for these programs to be offered to fathers. The effectiveness of interventions to reduce mental health problems in fathers was found to be mixed in two linked reviews examining perinatal paternal mental health (Goldstein et al., 2020; Rominov et al., 2016).

Ten reviews examined the effectiveness of interventions to improve parenting capacity and/or parent–child relationship in families affected by parental mental health problems. Most reviews reported a positive effect including upon responsiveness and skill of the parent (Barlow et al., 2021; Basogul & Buldukoglu, 2015; Letourneau et al., 2017) and child development (Basogul & Buldukoglu, 2015; Leijten et al., 2019; Letourneau et al., 2017; MacBeth et al., 2015). However, reviews found limited evidence of effect upon parent–child relationships (Barlow et al., 2016; Huang et al., 2020; Rayce et al., 2020), with only two showing effect (Jidong et al., 2021; Tsivos et al., 2015). The components of effective interventions included dyadic psychological interventions, home visiting programs, parent therapy, skills training, and mentalization-based intervention therapy.

##### Intimate Partner Violence and Abuse

Six reviews examined parenting capacity interventions for IPVA, providing evidence to suggest that interventions addressing parenting skills in mothers impacted by IPVA may have positive impacts upon parents and children. Anderson and van Ee (2018) found that a multileveled program of mothers and children working both separately and jointly together across psychosocial sessions might generate the most successful psychosocial recovery for mothers and children who have experienced violence in the home. The mechanism by which this happens is likely to be, they concluded, a collaborative one, focused on enhancing the dyadic interaction between mothers and their children.

Ryan and Roman (2019) reviewed six family-centered parenting interventions which included conflict resolution and communication skills, including knowledge and awareness-raising of family violence. Interventions were community-based group sessions, delivered to families, and most involved families from low-socioeconomic circumstances. They aimed to assist families to reduce violence and minimize the effects of IPVA, such as depression in parents and behavioral misconduct in children. The authors reported successful long-term outcomes, including a reduction in incidents of IPVA and reduced trauma symptoms of mothers. The review also found reduced child externalizing behaviors and increased pro-social activities among children and concluded that family-centered approaches facilitate long-term success especially in comparison to interventions targeted only for the perpetrator.

A further review undertook 6 meta-analyses of 21 studies of trauma-informed parenting interventions delivered to parents (mothers and fathers) and children, to better understand their potential impact on parenting practices, as well as child outcomes after exposure to IPVA (Lindstrom Johnson et al., 2018). The authors found that trauma-informed parenting interventions are effective at increasing positive parenting practices (SMD: 0.72, 95% CI [0.43, 1.00]) as well as reducing internalizing problems (SMD: 0.59, [0.43, 0.74]), externalizing problems (SMD: 0.48, [0.34, 0.62]), and trauma symptoms amongst children.

One review (Rizo et al., 2011) identified parenting interventions delivered separately to mothers, fathers, and to mothers and children respectively, all of which were described as having positive effects on parenting, but did not report effect sizes. Interventions for mothers sought to increase parenting efficacy, as well as enhance self-acceptance and well-being; these demonstrated improvements in parental self-efficacy and emotional well-being. Further, children who participated in the child–caregiver parenting intervention showed improvements in compliance as well as behavior problems. Common components identified across these interventions included CBT focused on addressing traumatic symptoms/experiences, parent sessions which integrated skills training in the context of trauma, as well as social support and coping skills, and joint sessions aimed at improving parent/child interactions.

Austin et al. (2019) reported on interventions which incorporated psychotherapy and parent training in the context of IPVA for women and children. They concluded that due to the heterogeneity of the existing interventions and the limitations of the research base, it was not clear which interventions were most effective in addressing the needs of women parenting in the context of IPVA. Bilukha et al. (2005) conducted a systematic review of the effectiveness of early childhood home visitation for preventing violence. These visitations were targeted to the whole family, and provided to specific target groups, such as low-income households; minorities; young parents; less educated; first-time mothers; substance abusers; or children at risk of abuse or neglect. The authors found an absence of evidence to determine the effectiveness of early childhood home visitation in preventing violence by parents or intimate partner violence in visited families.

##### Parental Substance Use

One review by R. McGovern, Newham, et al. (2021) found integrated interventions for parents which combined both parenting- and substance use-targeted components may be effective at reducing alcohol use (SMD: −0.56, [−0.96, −0.16]) and drug use (SMD: −0.39, [−0.75, −0.03]). They cautioned that a parenting intervention only, which does not incorporate an adjunctive substance use component, may not reduce frequency of substance use. R. McGovern, Newham, et al. (2021) also found that parents may be better able to reduce their substance use if children were not present in the sessions. Interventions also appeared to be more often beneficial for fathers than for mothers.

Two further reviews reported on substance use levels following integrated interventions, which incorporate substance use treatment alongside pregnancy, parenting, or child services; both showed a reduction in maternal substance use. Moreland and McRae-Clark (2018) reported that overall substance use in parents significantly decreases following engagement in a parenting intervention in such integrated substance use treatment programs (but did not report effect sizes). Milligan et al. (2010) also found that integrated programs are effective in reducing maternal substance use in comparison to no-treatment (alcohol use: SMD: 0.40, [−0.31, 0.48] and drug use: SMD: 0.65, [0.57, 0.74]), but were not significantly more effective than non-integrated programs.

Two linked reviews also explored integrated programs for mothers. While these reviews did not report on outcomes for parental substance use, Niccols et al. (2010) concluded integrated programs are associated with a small advantage over non-integrated services in improving maternal mental health outcomes. Furthermore, Niccols et al. (2012) found that integrated programs are associated with a small advantage over addiction treatment-as-usual in parenting skills outcomes. A further review reported that integrated interventions, incorporating substance use treatment, and family counseling or parenting skills training, were positively associated with the likelihood of family reunification (Murphy et al., 2017).

Results were mixed as to whether parenting interventions for parents who use substances resulted in improved parenting skills or child outcomes. Five reviews identified trials which intervened to enhance the parenting skills of the parent who uses substances and reported positive outcomes (Bowie, 2004; R. McGovern, Newham, et al., 2021; Peisch et al., 2018; West et al., 2020), other reviews reported mixed results (Bowie, 2004; Calhoun et al., 2015; Krahn et al., 2018; Peisch et al., 2018) and low quality evidence (West et al., 2020). Further, Calhoun et al. (2015) found that interventions which focus on improving parenting practices and family functioning may be effective in reducing problems in children affected by parental substance abuse, with effect sizes ranging from SMD 0.20 to 0.50.

##### Psychological and Supportive Interventions for Children Affected by Parental Risk Factors of Mental Health Problems, IPVA, and/or Substance Use

Six reviews examined psychological and/or supportive interventions to reduce mental health problems in children and adolescents of mentally ill parents. Psychological interventions such as CBT and IPT for children/adolescents were found to be effective, and resulted in significant small effects for global psychopathology (SMD: 0.13), as well as internalizing symptoms (SMD: 0.17) (Thanhäuser et al., 2017). Similarly, a meta-analysis of psychoeducational, family communication, and CBT interventions (Loechner et al., 2018) reported that preventative effects on the reduction of depressive/internalizing symptoms in children were small but significant at post-intervention for children of parents with mental illness (SMD: −0.20, [−0.34, −0.06]). Siegenthaler et al. (2012) identified 13 randomized controlled trials of preventive interventions for children of mentally ill parents. The aim of these interventions, delivered to adolescents, was to increase their knowledge and understanding of parents’ mental disorders and to strengthen their resilience. Meta-analysis indicated that the risk of developing the same mental illness as the parent following intervention was decreased by 40%. Interventions were effective at reducing children’s internalizing (SMD: −0.22, [−0.37, −0.08]) and externalizing (SMD: −0.16, [−0.36, 0.04]) problems. Similarly, Havinga et al. (2021) performed a meta-analysis of interventions which combined psychoeducational elements with skills training and/or cognitive behavioral therapy elements for children of depressed parents. The review reported reduced symptom levels in offspring at post-intervention (SMD: −0.19, [−0.36, −0.02]), maintained at 12-month follow-up. Reviews of educational interventions and those which sought to develop coping skills in children were found to have no effect (Basogul & Buldukoglu, 2015; Fraser et al., 2006).

Only one review examined interventions for the individual child affected by IPVA. Rizo et al. (2011) examined effectiveness of interventions that, either directly or indirectly, target children exposed to IPVA. The authors identified interventions including counseling and crisis/outreach which focused solely on children. Post-intervention children showed improvements in behavior problems, self-esteem/self-concept, attitudes, and knowledge related to anger and violence, anxiety, depression, aggression, social competence, emotional difficulties, trauma symptoms, and knowledge of resources and safety. No reviews examined the effect of psychological or supportive interventions for children affected by parental substance use.

##### Interventions for Children Affected by Multiple Parental Risk Factors

Two reviews examined interventions for children affected by a combination of parental risk factors including parental mental health problems, IPVA, and substance use. Marie-Mitchell and Kostolansky (2019) reviewed 20 intervention studies which combined parenting education, social service referrals, and social support for families of children aged 0 to 5 years affected by a variety of parental risk factors, including parental IPVA, mental health problems, and substance use. Eight of 15 studies that measured child health outcomes, and 15 of 17 studies that assessed the parent−child relationship, demonstrated improvement. The review authors concluded that multicomponent interventions, in particular those which utilize professionals to provide high-intensity home support, are effective at reducing the impact of childhood adversities on child behavioral/mental health problems and improving parent−child relationships for young children. In the only review that examined solely interventions offering material support, Courtin et al. (2019) identified 28 upstream interventions such as income supplementation and maintenance, welfare reform, conditional and unconditional cash transfers, health insurance, and cash/food vouchers. These interventions were often means-tested and targeted at low-income households, and in some instances at particular at-risk groups such as young mothers. Thirty-five percent of the reviewed socioeconomic interventions reported reductions in exposure to adverse childhood experiences. The review indicated that effect sizes were modest for adverse parenting (ranging from SMD 0.04 to −0.10) and household mental illness (ranging from SMD 0.001 to −0.13). Effect sizes for exposure to IPVA ranged from SMD 0.001 to −0.47, and for substance use the reported effect size was SMD 0.49. Housing, conditional cash transfer, and income supplementation interventions were the most promising interventions identified by the review, reducing exposure to adversity by 50%, 42%, and 33% respectively. The authors noted that some interventions were associated with adverse outcomes, such as increased substance use and family dissolution, potentially explained by increased independence from women who were the recipients of cash transfers. The authors concluded that overall, the current evidence suggests that upstream interventions contribute to the reduction of adverse childhood experiences and their potential impacts upon parents and children, but stressed that such interventions should complement psychosocial programs, for example, to develop children’s resilience to family adversities.

## What Is the Evidence Around the Experiences of Interventions?

Five reviews explored experiences of interventions. Alves et al. (2018) undertook a qualitative systematic review of 24 partner-inclusive interventions aimed at preventing and treating women’s PND. The content of the sessions was largely based upon psychoeducation around PND and parenthood and coping strategies, such as emotional and practical support by partners, to facilitate the transition to parenthood. The focus of the review was the effect of partner’s participation on the women’s response to the interventions. Qualitative accounts of participants emphasized the importance of their partner’s inclusion in postpartum depression interventions. However, the authors of the review concluded that scarce information about the attendance rates of partners made it difficult to determine if the partner’s participation was associated with the intervention’s efficacy.

Leonard et al. (2018) examined qualitative literature surrounding family-focused practice and home visiting for mothers and their families affected by maternal mental illness and substance use. They identified the key themes from these interventions as a need for mothers to have a reliable and flexible service; the ambiguity and differing interpretations of mental illness in home visiting needs to be addressed; and the need to take a more holistic view of the family unit rather than the current focus solely on mothers. Howarth et al. (2019) provided a qualitative synthesis on experiences of receiving IPVA interventions with the aim of identifying factors at different levels of the social–ecological context that may influence parent and child readiness to take up child-focused interventions. They concluded that such readiness may differ from readiness to take up safety-promoting behaviors and requires knowledge and awareness of the impacts of IPVA on the child. Parental support was also found to be important in facilitating children’s involvement in a therapeutic intervention.

Sword et al. (2009) reported women’s perceptions of benefits for themselves and their children of integrated treatment programs, including substance use treatment and a parenting support service. The authors identified the presence of children in treatment as a motivating factor for mothers to remain in the programs. Women also perceived the outcomes of participating in an integrated intervention program were sustained sobriety or decreased substance use, enhanced capacity for parenting, and improved maternal–child communication and relationships. Additionally, Usher et al. (2015) reported parent and children’s perceptions of family-based interventions for children of parents who use substances. The authors found that opportunities for positive parent–child interactions, supportive peer-to-peer relationships, and knowledge of addiction and its impacts accounted for effective interventions leading to improvements in family functioning and positive child psychosocial outcomes.

## Discussion

The findings of this review of 62 reviews suggest that despite a large volume of research into interventions that address the risk factors of interest in isolation, there is limited evidence for the effectiveness of interventions for families with children who experience a combination of risks, for example parental IPVA, mental health problems, and substance use. This is an important issue since these risk factors are known to commonly co-occur and impact upon on each other in a syndemic manner (Lacey et al., 2022; Lanier et al., 2018). Most intervention evaluation has focused on mothers, and particularly considered the perinatal period and mental health outcomes. There were only two reviews of interventions to address the structural or upstream factors such as poverty which compound these parental risk factors (Adjei et al., 2021) and interact in syndemic ways.

The evidence from this review indicates that families affected by parental mental health may be best served by integrated interventions which combine therapeutic interventions for the parent alongside parent skills training. It is not clear from the evidence however, whether parents and children should receive interventions together or separately (or a combination of both). Simply addressing the parenting risk factor and improving parenting might not be enough to improve outcomes for children, however. It is likely that children require psychological intervention to help them to overcome the impact of exposure to adversity (R. McGovern, Smart, et al., 2021). This child-focused intervention is likely to be separate from the parent and provide support for them and their needs directly. CBT and IPT in particular show promise for children of parents with mental ill-health; however, there is a paucity of evidence for those affected by parental IPVA and substance use.

The majority of included reviews addressing IPVA are focused on mothers and incorporate interventions that seek to support women who have experienced violence from male partners, and are aimed at protecting children from what is most likely paternal violence and aggression. Within the reviewed literature, there is a focus on men as perpetrators of violence within the family, and little acknowledgement of reciprocally violent partners or abusive women. Nonetheless, despite this focus on women as victims, there is often less focus on women’s mental well-being, and instead individualizing approaches place responsibility onto often vulnerable mothers which can contribute to “victim-blaming” (Mowat & Macleod, 2019). This approach may not take account of complex dynamics, where for example economic constraints, and perceived loss of control, may influence physical violence or coercive control by fathers (living with or away from the family) and contribute to poor mental health and/or substance use as a coping mechanism which compound difficulties in parenting. Moreover, abusive fathers can be repositioned as a problem for mothers for which they are responsible (Callaghan, 2015), and the discourse that mothers who experience violence have “failed to protect” their children places the burden not upon the perpetrator, but upon the mother. It is then her responsibility to learn to parent in different ways, and such an approach misses the complex implications that IPVA has upon families, and in particular the relationships between mothers and their children (Katz, 2019).

There is limited evidence of interventions for any of the single risk factors which attempt to tackle these risks at the wider family level—where parenting interventions are employed, these are again often targeted at mothers or at mothers and their children. There were very few reviews focused on paternal mental health, and where interventions which addressed the mental health of fathers were reviewed, the evidence was equivocal. Further, not all of the “family-focused” interventions targeted or engaged with fathers, but rather addressed paternal well-being indirectly by focusing on the mother, infant, or couple relationship. Fathers or partners are also often absent from interventions to address maternal mental health problems. Interventions therefore may need to be designed and tailored for fathers (DeGarmo, 2020) and these designs should consider the nature of the relationship between IPVA, mental health, and substance use (Stephens-Lewis et al., 2021).

Where interventions did address multiple risk factors, the review evidence indicates these approaches may be effective. Multicomponent interventions were found to be effective at reducing the impacts of adversities on child behavioral and mental health outcomes, as well as improving parent–child relationships (Marie-Mitchell & Kostolansky, 2019). Based on the above, the evidence would suggest that families facing adversities may be best served by healthcare professional-led interventions that involve home visits over a sustained period, and which include connection to community-based services, as well as supporting parenting capacity and skills, based upon parental need (Lowell et al., 2011). Evaluations of the longer-term impacts of interventions would also be a beneficial addition to the evidence.

Similarly, upstream interventions such as income supplementation and welfare reform were demonstrated to reduce a variety of adverse childhood experiences and their impacts upon parents and families (Courtin et al., 2019; Marie-Mitchell & Kostolansky, 2019). Together, these findings strengthen the argument for a syndemic approach to understanding and addressing family adversity. Material support for families facing adversities alongside poverty are seemingly absent from the vast majority of interventions identified within this review of reviews. Support services and interventions have previously been criticized for rarely engaging effectively with the impact of income, employment, and housing conditions on families and children, and this failure to recognize the difficulties parents may face in meeting children’s needs compounds this harm, as well as feelings of shame and stigma (Bywaters et al., 2022). Any intervention or policy approach that ignores the socioeconomic context of family adversity is therefore flawed. In order to ameliorate adverse health and behavioral outcomes in children and families, policies which address upstream drivers of poor health, and which seek to tackle the synergistic interaction of two or more coexisting risk factors are required (Adjei et al., 2021; Walsh et al., 2019). This is especially pertinent at a time when a cost of living crisis presents extra stress for families, with potential impacts upon health (Iacobucci, 2022).

This review further strengthens the call for intervention development to be informed by children and young people who have themselves experienced such adversities, to ensure it addresses their ongoing and multi-faceted needs (Lorenc et al., 2020). Extensive qualitative evidence indicates the need for longer-term interventions which allow the necessary time to build up trust and address the needs of children and young people affected by family adversity (Lester et al., 2019).

## Strengths and Limitations

This review provides a comprehensive and high-level view of the available evidence in a broad and complex area, and identifies important gaps in the literature; however, the methodology creates challenges and has limitations. Challenges encountered include an overlap between reviews; the quality and inconsistency of reporting within reviews; and synthesizing heterogeneous findings (Pollock et al., 2017). This methodology can involve double counting of primary studies, and while we checked primary studies for their relevance to the review question, we only extracted data from the systematic reviews. However, given the high-level nature of the synthesis, double counting of interventions is unlikely to have a major impact on the interpretation of findings. While a strength of the methodology is the ability to efficiently synthesize the highest levels of evidence across a breadth of literature, the output of the review is limited by the content of the included reviews and a potential lack of precision. Further, interventions focused on individuals and families are arguably easier to implement and evaluate than large-scale trials or policy interventions, which may explain their greater prevalence in the reviews identified here. Larger-scale, system-level interventions are more difficult to implement and to demonstrate effectiveness, and additionally any evaluations of such interventions may not have been captured in the selected research databases. Given the high volume of reviews, we were also unable to perform a detailed search or synthesis of gray literature. The evidence surrounding cumulative risks and syndemic approaches is also relatively recent, and this therefore may reflect the scarcity of such approaches in the published literature. Further research is required into the precise mechanisms and common components of those interventions which have been identified as effective within this review.

## Conclusions

The evidence for interventions for vulnerable families who are exposed to parental IPVA, mental health problems, and substance use is equivocal, with significant gaps. The strongest available evidence suggests that CBT to address perinatal mental health problems in combination with social/financial support may be effective. While there is some evidence that integrated interventions may be effective for parents, addressing parental risk factors and improving parenting capacity may not be enough to improve outcomes for children. It is likely that children require psychological intervention themselves to help them to overcome the impact of exposure to adversity.

Most intervention approaches focus on mitigating individual psychological harms and seek to address risk factors in isolation. These interventions may not therefore address the cumulative impacts of syndemic, co-occurring risks, or the social factors that may compound adversities. To ameliorate adverse health and behavioral outcomes in children and families associated with parental mental health, substance use, and IPVA, policies which address upstream drivers of poor health, and which seek to tackle the synergistic interaction of these coexisting risk factors are required.

## Implications for Practice, Policy, and Research

*Practice*

● Treating individuals and intervening with parental risk factors in isolation may not be suitable for families with complex needs.

● Practitioners should adopt poverty and trauma-informed practices to address these needs.

*Policy*

● Policies such as income supplementation, and early years provision for child mental health for example, may help to address upstream drivers of poor health such as stress and poverty within families.

● Policies should recognize the synergistic interaction of coexisting risk factors in order to ameliorate adverse outcomes for families and children.

*Research*

● More evidence is required on interventions for fathers and older children; as well as evaluations of interventions which seek to address syndemic, co-occurring risks.

● Interventions should be informed by those with lived experience, for example by incorporating qualitative methods into research designs, and by public and patient involvement in the co-designing and co-production of interventions.

## Supplemental Material

sj-docx-1-tva-10.1177_15248380231153867 – Supplemental material for Interventions to Reduce Parental Substance Use, Domestic Violence and Mental Health Problems, and Their Impacts Upon Children’s Well-Being: A Systematic Review of Reviews and Evidence Mapping Click here for additional data file. Supplemental material, sj-docx-1-tva-10.1177_15248380231153867 for Interventions to Reduce Parental Substance Use, Domestic Violence and Mental Health Problems, and Their Impacts Upon Children’s Well-Being: A Systematic Review of Reviews and Evidence Mapping by Simon Barrett, Cassey Muir, Samantha Burns, Nicholas Adjei, Julia Forman, Simon Hackett, Raeena Hirve, Eileen Kaner, Rebecca Lynch, David Taylor-Robinson, Ingrid Wolfe and Ruth McGovern in Trauma, Violence, & Abuse

sj-docx-2-tva-10.1177_15248380231153867 – Supplemental material for Interventions to Reduce Parental Substance Use, Domestic Violence and Mental Health Problems, and Their Impacts Upon Children’s Well-Being: A Systematic Review of Reviews and Evidence Mapping Click here for additional data file. Supplemental material, sj-docx-2-tva-10.1177_15248380231153867 for Interventions to Reduce Parental Substance Use, Domestic Violence and Mental Health Problems, and Their Impacts Upon Children’s Well-Being: A Systematic Review of Reviews and Evidence Mapping by Simon Barrett, Cassey Muir, Samantha Burns, Nicholas Adjei, Julia Forman, Simon Hackett, Raeena Hirve, Eileen Kaner, Rebecca Lynch, David Taylor-Robinson, Ingrid Wolfe and Ruth McGovern in Trauma, Violence, & Abuse
