# Supplementary material for: Interventions to Reduce Parental Substance Use, Domestic Violence and Mental Health Problems, and Their Impacts Upon Children’s Well-Being: A Systematic Review of Reviews and Evidence Mapping
Source: Trauma Violence Abuse. 2023 Feb 15;25(1):393–412. doi: 10.1177/15248380231153867 (PMC10666514; doi:10.1177/15248380231153867)
Supplement: sj-docx-1-tva-10.1177_15248380231153867 – Supplemental material for Interventions to Reduce Parental Substance Use, Domestic Violence and Mental Health Problems, and Their Impacts Upon Children’s Well-Being: A Systematic Review of Reviews and Evidence Mapping [file sj-docx-1-tva-10.1177_15248380231153867.docx]

ProQuest Search Strategy

17 March 2021

(TI((systematic OR state-of- the-art OR scoping OR literature OR umbrella AND review* OR overview* OR assessment* OR review* of reviews OR meta- analy* OR metaanaly* OR research evidence OR metasynthe* OR meta-synthe*) AND (Interven* OR intervention study OR Randomised Control Trial OR RCT OR prevent* Interven* OR intervention study OR Randomised Control Trial OR RCT OR prevent* OR Social polic* OR health polic* OR public health intervention* OR socioeconomic intervention* OR Social care OR social work OR welfare service* OR social services OR child welfare OR family service* OR family support OR family program OR Psychosocial intervention* OR psychological intervention* OR family-focused OR relationship-based intervention* OR family based intervention OR program*) AND (Parent* OR mother OR maternal OR father OR paternal OR famil* OR Child* OR infant* OR adolescent* OR adolescence OR teen* OR youth OR young pe* OR young adult)) AND ab((Mental health OR mental ill-health OR Mental illness OR mentally ill OR mental disorder* OR depress* OR anxiety OR pyschiat* OR wellbeing OR well-being OR quality of life OR self-esteem OR self perception OR post traumatic stress disorder OR PTSD OR Obsessive Compulsive disorder OR OCD OR panic disorder) OR (Domestic violence OR domestic abuse OR perpetrator* OR intimate partner violence OR emotional abuse OR psychological abuse OR verbal abuse OR economic abuse OR Financial abuse OR Intimidat* OR Isolat* OR control* OR coerc*) OR (substance-related disorders OR alcohol-related disorders OR amphetamine-related disorders OR cocaine-related disorders OR inhalant abuse OR marijuana abuse OR opioid-related disorders OR phencyclidine abuse OR psychoses OR substance-induced OR substance abuse OR intravenous OR substance withdrawal syndrome OR alcohol withdrawal OR stimulant* OR polydrug* OR drug* OR substance abus* OR dependen* OR addict* OR disorder* OR intoxicat* OR misuse* OR alcohol OR dependen* OR drink* OR intoxicat* OR abus* OR misus* OR risk* OR consum* OR excess* OR reduc* OR intervention* OR drink* AND excess OR heavy OR heavily OR harm OR harmful OR hazard* OR risky OR binge OR harmful OR problem*))) AND (stype.exact("Scholarly Journals") AND PEER(yes))
