# Supplementary material for: Interventions to Reduce Parental Substance Use, Domestic Violence and Mental Health Problems, and Their Impacts Upon Children’s Well-Being: A Systematic Review of Reviews and Evidence Mapping
Source: Trauma Violence Abuse. 2023 Feb 15;25(1):393–412. doi: 10.1177/15248380231153867 (PMC10666514; doi:10.1177/15248380231153867)
Supplement: sj-docx-2-tva-10.1177_15248380231153867 – Supplemental material for Interventions to Reduce Parental Substance Use, Domestic Violence and Mental Health Problems, and Their Impacts Upon Children’s Well-Being: A Systematic Review of Reviews and Evidence Mapping [file sj-docx-2-tva-10.1177_15248380231153867.docx]

Supplementary Table: Quality appraisal of reviews using the Joanna Brigg’s Institute Critical Appraisal Checklist for Systematic Reviews and Research Syntheses tool. Key: Yes (🗸), No (X), Unclear (?), Not applicable (–).

| **First Author (year)** | **Aim** | **Inclusion criteria** | **Search Strategy** | **Sources/ resources** | **Study appraisal** | **Two (+) reviewers** | **Data extraction** | **Methods** | **Publication bias** | **Policy/ practice** | **New research** |
| --- | --- | --- | --- | --- | --- | --- | --- | --- | --- | --- | --- |
| **Parental mental health problems** | | | | | | | | | | | |
| Alexander (2018) | 🗸 | 🗸 | 🗸 | 🗸 | 🗸 | 🗸 | 🗸 | 🗸 | 🗸 | 🗸 | 🗸 |
| Alves (2018) | 🗸 | 🗸 | 🗸 | 🗸 | 🗸 | 🗸 | 🗸 | 🗸 | ? | 🗸 | 🗸 |
| Barlow (2003) | 🗸 | 🗸 | 🗸 | 🗸 | 🗸 | 🗸 | 🗸 | 🗸 | 🗸 | 🗸 | 🗸 |
| Barlow (2012) | 🗸 | 🗸 | 🗸 | 🗸 | 🗸 | 🗸 | 🗸 | 🗸 | 🗸 | 🗸 | 🗸 |
| Barlow (2016) | 🗸 | 🗸 | 🗸 | 🗸 | 🗸 | 🗸 | 🗸 | 🗸 | 🗸 | 🗸 | 🗸 |
| Barlow (2021) | 🗸 | 🗸 | 🗸 | 🗸 | 🗸 | 🗸 | 🗸 | 🗸 | 🗸 | X | X |
| Basogul (2015) | 🗸 | 🗸 | 🗸 | 🗸 | X | ? | ? | 🗸 | X | X | X |
| Fraser (2006) | 🗸 | 🗸 | 🗸 | 🗸 | 🗸 | 🗸 | 🗸 | 🗸 | 🗸 | 🗸 | 🗸 |
| Daley (2009) | 🗸 | 🗸 | 🗸 | 🗸 | 🗸 | 🗸 | 🗸 | 🗸 | X | X | X |
| *Goldstein (2020) | 🗸 | 🗸 | 🗸 | 🗸 | 🗸 | X | 🗸 | 🗸 | X | – | – |
| Hanach (2021) | 🗸 | 🗸 | 🗸 | 🗸 | 🗸 | 🗸 | 🗸 | 🗸 | 🗸 | – | 🗸 |
| Havinga (2021) | 🗸 | 🗸 | 🗸 | 🗸 | 🗸 | X | 🗸 | 🗸 | X | 🗸 | 🗸 |
| Huang (2020) | 🗸 | 🗸 | 🗸 | 🗸 | 🗸 | 🗸 | 🗸 | 🗸 | 🗸 | – | 🗸 |
| Jidong (2021) | 🗸 | 🗸 | 🗸 | 🗸 | 🗸 | 🗸 | 🗸 | 🗸 | ? | 🗸 | 🗸 |
| Kim (2019) | 🗸 | 🗸 | 🗸 | 🗸 | 🗸 | 🗸 | 🗸 | 🗸 | 🗸 | 🗸 | 🗸 |
| Lapp (2010) | X | ? | 🗸 | 🗸 | ? | ? | ? | 🗸 | ? | 🗸 | ? |
| Leger (2015) | 🗸 | 🗸 | 🗸 | 🗸 | 🗸 | X | X | 🗸 | X | 🗸 | 🗸 |
| Leijten (2019) | 🗸 | 🗸 | 🗸 | 🗸 | 🗸 | 🗸 | 🗸 | 🗸 | 🗸 | 🗸 | 🗸 |
| Leis (2009) | ? | 🗸 | 🗸 | 🗸 | ? | X | ? | 🗸 | X | 🗸 | 🗸 |
| **Leonard (2018) | 🗸 | 🗸 | 🗸 | 🗸 | 🗸 | 🗸 | 🗸 | 🗸 | X | 🗸 | 🗸 |
| **Leonard (2021) | 🗸 | 🗸 | 🗸 | 🗸 | 🗸 | 🗸 | 🗸 | 🗸 | 🗸 | 🗸 | 🗸 |
| Letourneau (2017) | 🗸 | 🗸 | 🗸 | 🗸 | 🗸 | 🗸 | 🗸 | 🗸 | 🗸 | – | 🗸 |
| Loechner (2018) | 🗸 | 🗸 | 🗸 | 🗸 | 🗸 | 🗸 | 🗸 | 🗸 | 🗸 | 🗸 | 🗸 |
| MacBeth (2015) | 🗸 | 🗸 | 🗸 | 🗸 | 🗸 | 🗸 | 🗸 | 🗸 | 🗸 | 🗸 | 🗸 |
| Morrell (2006) | 🗸 | 🗸 | 🗸 | 🗸 | 🗸 | 🗸 | ? | 🗸 | X | ? | X |
| Nair (2018) | 🗸 | 🗸 | 🗸 | 🗸 | 🗸 | 🗸 | 🗸 | 🗸 | 🗸 | X | 🗸 |
| Nillni (2018) | 🗸 | 🗸 | 🗸 | 🗸 | ? | ? | ? | 🗸 | 🗸 | X | 🗸 |
| Olhaberry (2013) | X | 🗸 | 🗸 | 🗸 | X | ? | X | 🗸 | X | 🗸 | 🗸 |
| Rayce (2020) | 🗸 | 🗸 | 🗸 | 🗸 | 🗸 | 🗸 | 🗸 | 🗸 | 🗸 | 🗸 | 🗸 |
| Reuveni (2020) | 🗸 | 🗸 | 🗸 | 🗸 | ? | ? | 🗸 | 🗸 | 🗸 | X | 🗸 |
| *Rominov (2016) | X | 🗸 | 🗸 | 🗸 | 🗸 | 🗸 | 🗸 | 🗸 | X | X | 🗸 |
| Shi (2017) | X | 🗸 | 🗸 | 🗸 | ? | 🗸 | ? | 🗸 | 🗸 | 🗸 | 🗸 |
| Siegenthaler (2012) | X | 🗸 | 🗸 | 🗸 | 🗸 | ? | ? | 🗸 | 🗸 | X | 🗸 |
| Song (2015) | 🗸 | 🗸 | 🗸 | 🗸 | 🗸 | 🗸 | 🗸 | 🗸 | 🗸 | 🗸 | 🗸 |
| Stevenson (2010) | 🗸 | 🗸 | 🗸 | 🗸 | 🗸 | 🗸 | 🗸 | 🗸 | X | 🗸 | 🗸 |
| Stiawa (2014) | 🗸 | 🗸 | 🗸 | 🗸 | ? | X | X | 🗸 | X | 🗸 | X |
| Thanhäuser (2017) | 🗸 | 🗸 | 🗸 | 🗸 | 🗸 | 🗸 | 🗸 | 🗸 | 🗸 | X | X |
| Tsivos (2015) | 🗸 | 🗸 | 🗸 | 🗸 | 🗸 | ? | ? | 🗸 | X | X | 🗸 |
| **Intimate partner violence and/or abuse** | | | | | | | | | | | |
| Anderson (2018) | 🗸 | 🗸 | 🗸 | 🗸 | 🗸 | 🗸 | 🗸 | 🗸 | 🗸 | 🗸 | 🗸 |
| Austin (2019) | 🗸 | 🗸 | 🗸 | 🗸 | ? | X | 🗸 | 🗸 | 🗸 | 🗸 | 🗸 |
| Bilukha (2005) | 🗸 | 🗸 | 🗸 | 🗸 | 🗸 | 🗸 | 🗸 | 🗸 | ? | 🗸 | – |
| Howarth (2019) | 🗸 | 🗸 | 🗸 | 🗸 | 🗸 | 🗸 | 🗸 | 🗸 | 🗸 | 🗸 | 🗸 |
| Lindstrom Johnson (2018) | 🗸 | 🗸 | 🗸 | 🗸 | 🗸 | 🗸 | 🗸 | 🗸 | 🗸 | 🗸 | 🗸 |
| Rizo (2011) | 🗸 | 🗸 | 🗸 | 🗸 | ? | ? | ? | 🗸 | X | 🗸 | 🗸 |
| Ryan (2019) | 🗸 | 🗸 | 🗸 | 🗸 | 🗸 | 🗸 | 🗸 | 🗸 | ? | X | X |
| **Parental substance use** | | | | | | | | | | | |
| Bowie (2004) | X | 🗸 | 🗸 | 🗸 | X | ? | X | 🗸 | X | 🗸 | 🗸 |
| Calhoun (2015) | 🗸 | 🗸 | 🗸 | 🗸 | 🗸 | 🗸 | 🗸 | 🗸 | 🗸 | 🗸 | 🗸 |
| Krahn (2018) | 🗸 | 🗸 | 🗸 | 🗸 | 🗸 | ? | 🗸 | 🗸 | 🗸 | – | 🗸 |
| McGovern, Newham (2021a) | 🗸 | 🗸 | 🗸 | 🗸 | 🗸 | 🗸 | 🗸 | 🗸 | 🗸 | 🗸 | 🗸 |
| McGovern, Smart (2021b) | 🗸 | 🗸 | 🗸 | 🗸 | 🗸 | 🗸 | 🗸 | 🗸 | 🗸 | 🗸 | 🗸 |
| ***Milligan (2010) | 🗸 | 🗸 | 🗸 | 🗸 | 🗸 | 🗸 | 🗸 | 🗸 | ? | 🗸 | 🗸 |
| Moreland (2018) | 🗸 | 🗸 | 🗸 | 🗸 | 🗸 | X | X | 🗸 | X | 🗸 | 🗸 |
| Murphy (2017) | 🗸 | 🗸 | 🗸 | 🗸 | 🗸 | ? | 🗸 | 🗸 | 🗸 | 🗸 | 🗸 |
| ***Niccols (2010) | 🗸 | 🗸 | 🗸 | 🗸 | 🗸 | 🗸 | 🗸 | 🗸 | ? | X | 🗸 |
| ***Niccols (2012) | 🗸 | 🗸 | 🗸 | 🗸 | 🗸 | 🗸 | 🗸 | 🗸 | X | X | 🗸 |
| Peisch (2018) | 🗸 | ? | 🗸 | 🗸 | X | ? | X | 🗸 | X | 🗸 | 🗸 |
| Sword (2009) | 🗸 | 🗸 | 🗸 | 🗸 | 🗸 | 🗸 | 🗸 | 🗸 | – | 🗸 | ? |
| Usher (2015) | 🗸 | 🗸 | 🗸 | 🗸 | 🗸 | ? | 🗸 | 🗸 | X | 🗸 | 🗸 |
| West (2020) | ? | 🗸 | 🗸 | 🗸 | 🗸 | 🗸 | 🗸 | 🗸 | 🗸 | X | 🗸 |
| **Parental substance use and intimate partner violence and/or abuse** | | | | | | | | | | | |
| Giusto (2018) | 🗸 | 🗸 | 🗸 | 🗸 | 🗸 | X | X | 🗸 | 🗸 | 🗸 | 🗸 |
| **All three parental risk factors** | | | | | | | | | | | |
| Courtin (2019) | X | 🗸 | 🗸 | 🗸 | 🗸 | 🗸 | 🗸 | 🗸 | 🗸 | 🗸 | 🗸 |
| Marie-Mitchell (2019) | 🗸 | 🗸 | 🗸 | 🗸 | 🗸 | ? | 🗸 | 🗸 | 🗸 | 🗸 | 🗸 |

*Note.* *Goldstein (2020) and Rominov (2016) are linked reviews. **Leonard (2018) and Leonard (2021) are linked reviews. ***Milligan (2010), Niccols (2010) and Niccols (2012) are linked reviews.
